# METTL14-regulated PI3K/Akt signaling pathway via PTEN affects HDAC5-mediated epithelial–mesenchymal transition of renal tubular cells in diabetic kidney disease

**DOI:** 10.1038/s41419-020-03312-0

**Published:** 2021-01-04

**Authors:** Zhaoxia Xu, Keqi Jia, Hui Wang, Feng Gao, Song Zhao, Fan Li, Jun Hao

**Affiliations:** 1 Department of Pathology, Hebei Medical University, Shijiazhuang, China; 2 Hebei Key Laboratory of Kidney Diseases, Shijiazhuang, China; 3 Center of Metabolic Diseases and Cancer Research, Institute of Medical and Health Science of Hebei Medical University, Shijiazhuang, China; 4 Department of Pathology, The Third Hospital of Hebei Medical University, Shijiazhuang, China

**Keywords:** Epithelial-mesenchymal transition, Insulin signalling, Diabetes complications

## Abstract

Histone deacetylase 5 (HDAC5) belongs to class II HDAC subfamily and is reported to be increased in the kidneys of diabetic patients and animals. However, little is known about its function and the exact mechanism in diabetic kidney disease (DKD). Here, we found that HDAC5 was located in renal glomeruli and tubular cells, and significantly upregulated in diabetic mice and UUO mice, especially in renal tubular cells and interstitium. Knockdown of HDAC5 ameliorated high glucose-induced epithelial–mesenchymal transition (EMT) of HK2 cells, indicated in the increased E-cadherin and decreased α-SMA, via the downregulation of TGF-β1. Furthermore, HDAC5 expression was regulated by PI3K/Akt signaling pathway and inhibition of PI3K/Akt pathway by LY294002 treatment or Akt phosphorylation mutation reduced HDAC5 and TGF-β1 expression in vitro high glucose-cultured HK2 cells. Again, high glucose stimulation downregulated total m6A RNA methylation level of HK2 cells. Then, m6A demethylase inhibitor MA2 treatment decreased Akt phosphorylation, HDAC5, and TGF-β1 expression in high glucose-cultured HK2 cells. In addition, m6A modification-associated methylase METTL3 and METTL14 were decreased by high glucose at the levels of mRNA and protein. METTL14 not METTL3 overexpression led to PI3K/Akt pathway inactivation in high glucose-treated HK2 cells by enhancing PTEN, followed by HDAC5 and TGF-β1 expression downregulation. Finally, in vivo HDACs inhibitor TSA treatment alleviated extracellular matrix accumulation in kidneys of diabetic mice, accompanied with HDAC5, TGF-β1, and α-SMA expression downregulation. These above data suggest that METTL14-regulated PI3K/Akt signaling pathway via PTEN affected HDAC5-mediated EMT of renal tubular cells in diabetic kidney disease.

## Introduction

Diabetic kidney disease (DKD) is a kind of severe chronic complication of diabetes mellitus (DM) and has been reported to have multiple morphologic changes including hypertrophy and proliferation of mesangial cells, epithelial–mesenchymal transition (EMT) of renal tubular cells, apoptosis of podocytes, and so on^1^. Among these, EMT of renal tubular cells is the most common reason to cause impaired renal function^2^. TGF-β1 pathway has been demonstrated to be the important pro-fibrosis factor in the pathogenesis of diabetic kidney disease^3^.

Accumulating evidence suggests that inhibition of histone deacetylase (HDAC) ameliorates diabetic kidney disease manifestations and phenotypes such as fibrosis, inflammation, cell death, and albuminuria^4,5^. A pan-HDAC inhibitor trichostatin A (TSA) was reported to suppress EMT, indicated in upregulation of E-cadherin and downregulation of collagen type I, in TGF-β1-treated human renal proximal tubular epithelial cells^6^. HDAC5 belongs to HDAC family that is found to regulate gene transcription by affecting histone acetylation and chromatin remodeling. Among four kinds of HDAC genes, HDAC5 belongs to class II HDAC subfamily, together with HDAC4, 6, 7, 9, and 10^7^. Recently, Wang and co-workers revealed the increased expression of HDAC2, HDAC4, and HDAC5 in the kidneys of STZ-diabetic rats and db/db mice, and increased expression of HDAC4 and HDAC5 in the kidneys of humans with diabetic kidney disease^8^. However, the effect and mechanism of HDAC5 on EMT of renal tubular cells of DN is still not known.

Phosphatidylinositol-3-kinase (PI3K), known as a lipid kinase, generates PIP3 that regulates the translocation of Akt to plasma membrane (PM) as a second messenger. Then, Akt is activated by phosphorylation modification of threonine 308 and serine 473^9^. PI3K/Akt signaling pathway plays a key regulatory role in the development of diabetic kidney disease. PI3K/Akt pathway is activated in renal tubular cells under the diabetic condition to regulate cell growth, EMT, and lipid metabolism^10,11^.

N6-methyladenosine (m6A) RNA methylation is an abundant and conservative RNA modification in mammals and is regulated by a series of “writer”, “eraser”, and “reader” proteins. METTL3–METTL14 complex catalyzes m6A modification of RNA as “writer” and FTO and ALKBH5 remove methyl from RNA as “eraser”. “Reader” proteins are responsible for recognizing m6A methylated transcripts to regulate mRNA processing, translation, and degradation^12^. Deficiency of m6A RNA methylation is associated with many kinds of diseases, such as cancer and diabetes mellitus^13^.

In the present study, we first revealed that HDAC5 expression was increased in renal tubular cells of diabetic mice, fibrosis region of unilateral ureteral obstruction (UUO) mice, and in vitro high glucose-cultured human renal tubular cell line (HK2). Knockdown of HDAC5 inhibited high glucose-induced EMT in HK2 cells via TGF-β1 downregulation. Again, blocking PI3K/Akt pathway in high glucose-treated HK2 cells reduced HDAC5 expression, followed by TGF-β1 downregulation. Furthermore, m6A RNA methylation level was reduced in high glucose-stimulated HK2 cells. The m6A demethylase inhibitor MA2 suppressed Akt phosphorylation and HDAC5 expression. Then, m6A RNA methylation-related genes’ detection revealed the downregulation of METTL3, METTL14, and FTO in high glucose-cultured HK2 cells. Overexpression of METTL14 not METTL3 reversed high glucose-activated Akt pathway and HDAC5 expression via PTEN. Finally, in vivo administration of TSA reduced HDAC5 expression and extracellular matrix (ECM) accumulation in the kidneys of diabetic mice.

## Materials and methods

### Materials

Primary antibody against HDAC5 (sc-133225) was purchased from Santa Cruz Biotechnology Inc. (Santa Cruz, CA, USA). Primary antibodies against PTEN (ET1606-43), E-cadherin (ER63312) and α-SMA (ET1607-53) were bought from HuaAn Biotechnology Co. (Hangzhou, Zhejiang, China). Antibodies against phospho-Akt (Ser 473) (4060), phospho-Akt (Thr 308) (13038), METTL3 (96391), and METTL14 (51104) were purchased from Cell Signaling Technology Co. (Beverly, MA, USA). Antibodies against Akt (ab8805) and TGF-β1 (ab215715) were from Abcam Co. (Cambridge, MA, USA). Antibody against FTO (27226-1-AP) was bought from Proteintech Group, Inc. (Rosemont, IL, USA). Antibody against β-actin (AC026) was from ABclonal Biotechnology Co. (Wuhan, Hubei, China). Lipofectamine^TM^ 3000 Transfection Reagent was purchased from Invitrogen Co. (Carlsbad, CA, USA). Immunohistochemistry kit was bought from Zhongshan Golden Bridge Technology Co. (Beijing, China). DyLight 594-labeled goat secondary antibody was bought from KPL Co. (Gaithersburg, MD, USA). PrimeScript^TM^ RT reagent Kit with gDNA Eraser, SYBR^Ⓡ^ Premix Ex Taq^TM^ II (Tli RNaseH Plus), PrimeSTAR^®^ GXL DNA Polymerase, and Dpn I were bought from Takara Co. (Otsu, Shiga, Japan). EpiQuik^TM^ m6A RNA Methylation Quantification Kit was bought from Epigentek Group Inc. (Farmingdale, NY, USA). Masson trichrome kit was from Baso Diagnostics Inc. (Zhuhai, Guangdong, China). 4′, 6-Diamidino-2-phenylindole dihydro-chloride (DAPI), Streptozotocin (STZ) and insulin were bought from Sigma Chemical Co. (St. Louis, MO). LY294002 and TSA were bought from MCE Co. (Monmouth Junction, NJ, USA). The pGenesil-1 plasmid was purchased from Jingsai Co. (Wuhan, Hubei, China) for shRNA plasmid construction. TGF-β1 recombinant protein and Akt expression plasmid were bought from Sino Biological Inc. (Beijing, China). METTL3 and METTL14 expression plasmids were bought from YouBio Co. (Changsha, Hunan, China). The ethyl ester form of meclofenamic acid 2 (MA2) was a gift from Professor Caiguang Yang of the Chinese Academy of Sciences Shanghai Institute of Materia Medica.

### Animals and groups

C57BL/6J mice, at 6–8 weeks of age, were purchased from Vital River Laboratory Animal Technology Company (Beijing, China) and were housed and handled according to the guidelines approved by Hebei Medical University Animal Care and Use Committee. PASS Sample Size Software was used to estimate sample size. Simple random sampling method was used for the group allocation of experimental mice and the detection was performed blindly. Twenty mice were randomly divided into three groups: normal mice group (N), diabetic mice group (DM), and diabetic mice administrated with TSA group (DM + TSA). Type 1 diabetic mice were made by intraperitoneally injecting streptozotocin (STZ, 150 mg/kg body weight). Three days later, mice with random blood glucose greater than 11.1 mmol/L were regarded as diabetic models. Mice were intraperitoneally injected with TSA at 0.5 mg/kg weight three times per week. After two months, all mice were sacrificed for the related detections. Also, twelve mice were randomly divided into two groups: sham group and unilateral ureteral obstruction (UUO) group. UUO mice were made according to the standard protocol. In detail, the animals were anesthetized with the injection of 10% chloral hydrate. A midline incision of abdomen was made and the left ureter was ligated at two points. Then the abdominal incision was stitched. The mice with the left ureter exposed but not ligated were regarded as sham mice. After two weeks, all animals were killed and the related detections were performed.

### Cell culture and groups

Human renal proximal tubular cells (HK2) were cultured with Dulbecco’s Modified Eagle Medium/ Nutrient Mixture F-12 Ham (DMEM/F12 3:1 mixture) containing 10% serum and, 1% penicillin and streptomycin at 37 °C. High glucose treatment was carried out using medium containing 40 mmol/L glucose for the indicated time (1 and 6 h for Akt detection, 48 h for HDAC5 detection). For cell transfection of pGenesil-1-HDAC5 plasmid for 48 h, HK2 cells were randomly divided into two groups: pGenesil-1 group and pGenesil-1-HDAC5 group. To elucidate the effect of Akt pathway on HDAC5 expression, high glucose-cultured HK2 cells were randomly divided into two groups: DMSO group and LY294002 (20 μmol/L) group. For Akt phosphorylation site mutation, HK2 cells were randomly divided into three groups for 48 h transfection: Akt wild-type plasmid-transfected group (Akt WT), Akt (S473A) plasmid-transfected group (Akt S473A), and Akt (S473D) plasmid-transfected group (Akt S473D). Again, to investigate the effect of MA2 on Akt pathway and HDAC5 expression, HK2 cells were randomly divided into high glucose plus DMSO group (DMSO) and high glucose plus 50 μmol/L MA2 group (MA2). For insulin function, high glucose-cultured HK2 cells were randomly divided into DMSO group (DMSO), MA2 group (MA2), and MA2 plus 2 μg/mL insulin group (MA2 + insulin). In addition, to elucidate the effect of METTL3 and METTL14 on Akt pathway and HDAC5 expression, high glucose-cultured HK2 cells were divided into pcDNA3.1 group, pcDNA3.1-METTL3 group, and pcDNA3.1-METTL14 group.

### Small hairpin RNA plasmid construction

The shRNA plasmids aimed at HDAC5 and METTL14 were constructed and named as pGenesil-1-HDAC5 and pGenesil-1-METTL14. The target sequences of human HDAC5 (NM_001015053.1) and METTL14 (NM_020961.4) were, respectively, CTGTTATTAGCACCTTTAAGAA and GCTAATGTTGACATTGACT TA. The protocol was performed to construct plasmid as described previously^14^. The plasmid was identified with the methods of Sal I enzyme digestion and sequencing.

### Akt mutant plasmid construction

Wild-type Akt plasmid pCMV3-Akt was purchased from Sino Biological Inc. Point mutant plasmids pCMV3-Akt (S473A) and pCMV3-Akt (S473D) were constructed by the method of PCR. The primers for pCMV3-Akt (S473A) and pCMV3-Akt (S473D) were as follows: pCMV3-Akt (S473A) forward: ACTTCCCCCAGTTCGCCTACTCGGCCAGCG; pCMV3-Akt (S473A) reverse: CGCTGGCCGAGTAGGCGAACTGGGGGAAGT; pCMV3-Akt (S473D) forward: ACTTCCCCCAGTTCGACTACTCGGCCAGCG; pCMV3-Akt (S473D) reverse: CGCTGGCCGAGTAGTCGAACTGGGGGAAGT. The mutant plasmid was amplified with the corresponding primers and PrimeSTAR^®^ GXL DNA Polymerase, using wild-type plasmid as template. Then PCR product was transferred into DH5α for clone selection after Dpn I digestion. Finally, the recombinant plasmids were identified by DNA sequencing.

### Plasmid transfection

Lipofectamine^TM^ 3000 was used to perform cell transfection and the detailed protocol was as follows. A 2.5 µg plasmid and 5 µL P3000 were mixed with 125 µL serum-free DMEM medium, and then mixed with an additional 125 µL serum-free DMEM medium containing 5 µL Lipofectamine™ 3000. Five minutes later, the mixture was added into HK2 cells in 6-well plates. After 48 h, the cells were collected and the related detections were performed.

### Protein extraction and western blot

Protein extraction was performed according to the method used in the previous research^15^. In detail, cells were washed with PBS and lysed for 30 min at 4 °C with 2.5 mM Tris–HCl (pH 7.5), 100 mM NaCl, 0.1% Triton X-100, 1% NP-40, 30 mM sodium phosphate (pH 7.4), 1 mM sodium orthovanadate, 10 μg/mL leupeptin, and 10 μg/mL aprotinin. Subsequently, the homogenate was centrifuged at 12,000*g* for 30 min at 4 °C and the supernatant containing protein was collected. Also, renal tissue protein was extracted following the same procedure. After protein quantitative assay, 30 μg total protein was separated on 10% SDS-PAGE gel and transferred onto PVDF membrane, followed by an incubation with 5% BSA at 37 °C for 1 h. Next, blots were washed with TBST and incubated with primary antibodies overnight at 4 °C. The next morning, blots were rinsed with TBST and incubated with horseradish peroxidase-conjugated secondary antibody for 1 h at room temperature. Bands were visualized using ECL detection reagents for blots incubation. For quantitative analysis, the integrated optical density (IOD) bands were evaluated with LabWorks software (UVP Laboratory Products, Upland, CA, USA), normalized by β-actin.

### Real-time PCR

Total RNA was extracted from HK2 cells using Trizol reagent in accordance with standard procedure. The cDNA was synthesized using PrimeScript^TM^ RT reagent Kit with gDNA Eraser. Then SYBR^Ⓡ^ Premix Ex Taq^TM^ II (Tli RNaseH Plus) kit was used for real-time PCR detection in accordance with the protocol as follows: 95 °C for 5 min, 95 °C for 10 s, 55 °C for 30 s, 72 °C for 10 s, 40 cycles. Likewise, melting curves were made to confirm the amplification specificity. Primer sequences were as given in Table 1 and 18S was used to normalize the relative expression as an internal reference. The results were analyzed using the 2^−∆∆CT^ method and shown as relative quantity (gene/18S).

**Table 1:** Primers of human HDAC5, Fibronectin, Collagen 1, Collagen 3, FTO, METTL3, METTL14, PTEN, and 18S.

	Forward primer	Reverse primer	Product
HDAC5	TCTTGTCGAAGTCAAAGGAGC	GAGGGGAACTCTGGTCCAAAG	108 bp
Fibronectin	CGGTGGCTGTCAGTCAAAG	AAACCTCGGCTTCCTCCATAA	130 bp
Collagen 1	GAGGGCCAAGACGAAGACATC	CAGATCACGTCATCGCACAAC	140 bp
Collagen 3	GCCAAATATGTGTCTGTGACTCA	GGGCGAGTAGGAGCAGTTG	145 bp
FTO	ACTTGGCTCCCTTATCTGACC	TGTGCAGTGTGAGAAAGGCTT	145 bp
METTL3	TTGTCTCCAACCTTCCGTAGT	CCAGATCAGAGAGGTGGTGTAG	145 bp
METTL14	AGTGCCGACAGCATTGGTG	GGAGCAGAGGTATCATAGGAAGC	101 bp
PTEN	TTTGAAGACCATAACCCACCAC	ATTACACCAGTTCGTCCCTTTC	134 bp
18S	ATCCTCAGTGAGTTCTCCCG	CTTTGCCATCACTGCCATTA	106 bp

### Immunofluorescence

HK2 cells seeded on the cover slips in 6-well plate were fixed with 4% paraformaldehyde, permeabilized with 0.3% Triton X-100, and blocked with goat serum, in turn. Then, cells were incubated with primary antibodies overnight at 4 °C. After washing with PBS, cells were incubated with DyLight 594-conjugated secondary antibody at 37 °C for 2 h. After washing with PBS and counterstaing with DAPI, cells were observed and a photograph was taken under an inverted fluorescence microscope.

### Immunohistochemistry

HDAC5 expression in the kidneys of diabetic mice and UUO mice was detected by the method of immunohistochemistry. In detail, sections were boiled in pressure cooker for antigen retrieval, after deparaffinization and hydration. Afterwards, sections were treated using 0.3% H_2_O_2_ at room temperature for 10 min to quench endogenous peroxidase. Next, goat serum was used to incubate sections for 30 min at 37 °C to block non-specific staining. Then, sections were incubated with primary antibody overnight at 4 °C. After rinsing with PBS, sections were incubated with biotin-conjugated secondary antibody at 37 °C for 30 min and HRP-conjugated streptavidin at 37 °C for 30 min, in turn. Finally, sections were incubated with 3,3′‐diaminobenzidine tetrahydrochloride (DAB) to show the positive staining. PBS was used to replace primary antibody for negative control.

### Masson trichrome staining

Masson trichrome staining was applied to determine the ECM accumulation and fibrosis in the kidneys of diabetic mice and UUO mice, according to the protocol as follows. Renal tissue sections were deparaffinized, rehydrated, and immersed in Weigert iron haematoxylin solution for 10 min. Subsequently, rinsed with water, sections were immersed in Ponceau acid fuchsin solution for 10 min. Then sections were treated with phosphomolybdic-phosphotungstic acid for 10 min for differentiation. After immersed in aniline blue solution for 10 min, the sections were differentiated by treatment with 1% acetic acid for 1 min, followed by dehydration, clearing, and mounting.

### Total N6-methyladenosine RNA methylation detection

Total N6-methyladenosine RNA methylation was detected according to the instruction of EpiQuik^TM^ m6A RNA Methylation Quantification Kit. RNA extraction from HK2 cells was performed using Trizol reagent. First, the 80 μL BS (binding solution) was added to each well. Then RNA samples, negative control, and positive control were added into the designated wells and placed for 90 min at 37 °C. After washing with WB (washing buffer) for three times, 50 μL diluted CA (capture antibody) was added to each well and incubated for 60 min at room temperature. After rinsing with WB, 50 μL diluted DA (detection antibody) and 50 μL diluted ES (enhancer solution) were added into each well and incubated for 30 min at room temperature, in turn. Then, the 100 μL DS (developer solution) was used to incubate each well for 10 min to produce color change, followed by the supplement of 100 μL SS (stop solution) to stop the reaction. Finally, the absorbance was read on a microplate reader at 450 nm. The amount of m6A RNA of samples was calculated according to the standard curve. Then the percentage of m6A RNA methylation was the ratio of the amount of m6A RNA and the amount of input total RNA.

### Statistical analysis

All data were presented as mean ± SD from at least three independent experiments. GraphPad Prism 6 software was used for statistical analyses. For experiments with two groups, statistical analyses were performed using Student’s *t*-test. For more than two groups, statistical analyses were performed using one-way analysis of variance (ANOVA), followed by the Bonferroni post hoc test to determine the differences within and between groups. The Kruskal–Wallis test was used for the comparisons of data with non-normal distribution or heterogeneity of variance. *P* value <0.05 was considered as significant.

## Results

### HDAC5 expression was increased in renal tubular cells of diabetic mice and high glucose-treated HK2 cells

**Fig. 1:**
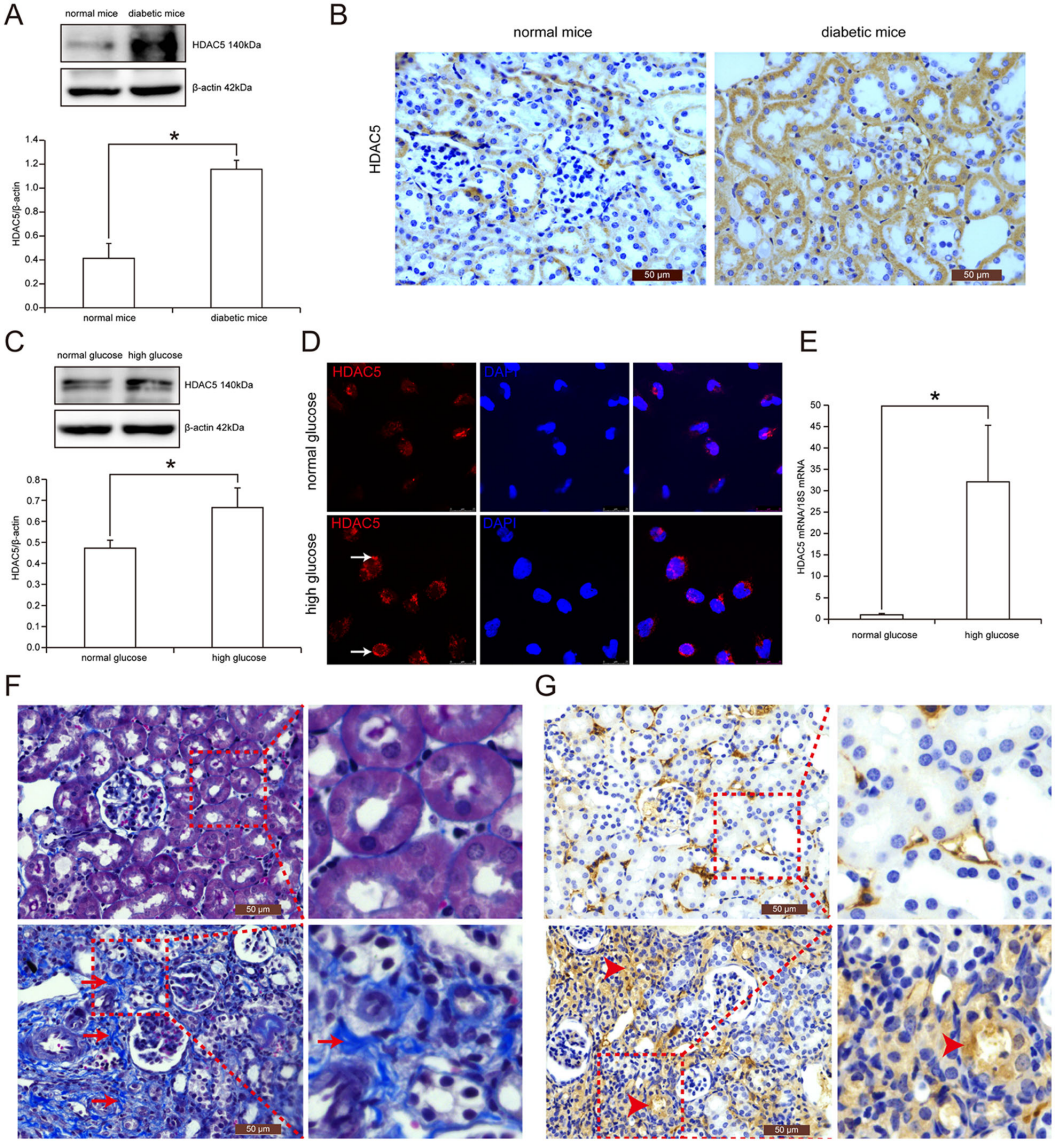
HDAC5 expression in renal tubular cells of diabetic mice and high glucose-treated HK2 cells. **A** Western blot and statistical analyses of HDAC5 expression in the kidneys of normal mice and diabetic mice. *n* = 6, *means *P* < 0.05 versus normal mice. **B** Immunohistochemistry of HDAC5 expression in the kidneys of normal mice and diabetic mice. **C** Western blot and statistical analyses of HDAC5 protein in HK2 cells treated with normal glucose and high glucose. Data were representative of three independent experiments. *Means *P* < 0.05 versus normal glucose group. **D** Immunofluorescence of HDAC5 expression in normal glucose-treated HK2 cells and high glucose-treated cells. White arrows show positive staining of HDAC5. **E** Real-time PCR of HDAC5 mRNA in HK2 cells treated with normal glucose and high glucose. Data were also representative of three independent experiments. *Means *P* < 0.05 versus normal glucose group. **F** Masson staining of extracellular matrix (ECM) accumulation in the kidneys of normal mice and unilateral ureteral obstruction (UUO) mice. Red arrows show ECM accumulation. **G** Immunohistochemistry of HDAC5 expression in the kidneys of normal mice and UUO mice (*n* = 6). Red triangles show positive expression of HDAC5.

We first detected the expression of HDAC5 in the kidneys of diabetic mice by the methods of western blot and immunohistochemistry. As seen in Fig. 1A, HDAC5 was increased by 2.80 times in the kidneys of diabetic mice compared with those of normal mice (*P* < 0.05). Furthermore, immunohistochemistry revealed that HDAC5 expressed in renal glomeruli, renal tubular cells, and renal interstitium. Especially, HDAC5 expression was enhanced in renal tubular cells of diabetic mice versus normal mice (Fig. 1B). Again, we explored the effect of high glucose known as the main feature of diabetes mellitus on HDAC5 expression in vitro-cultured human renal tubular cells (HK2 cells) and the results showed that HDAC5 was increased by 41.01% in HK2 cells treated with high glucose (40 mmol/L glucose) for 48 h compared to those treated with normal glucose (10 mmol/L glucose) (Fig. 1C). Similarly, immunofluorescence also confirmed the overexpression of HDAC5 in high glucose medium-cultured HK2 cells, indicated in green fluorescence in the cytoplasm of cells (Fig. 1D). In addition, HDAC5 mRNA was found to be upregulated by 31.15 times in high glucose-stimulated HK2 cells versus normal glucose-treated cells using real-time PCR technology (Fig. 1E). Interestingly, in unilateral ureteral obstruction (UUO) mice known as the classical renal fibrosis model, we found the prominently increased expression of HDAC5 in renal tubular cells located in fibrotic interstitium region, in comparison with those cells located in normal region (Fig. 1F, G). The above data suggested that HDAC5 expression was upregulated in renal tubular cells of diabetic kidney disease, which might be involved in epithelial–mesenchymal transition (EMT) of renal tubular cells and renal interstitial fibrosis.

### Knockdown of HDAC5 ameliorated high glucose-induced EMT of HK2 cells

**Fig. 2:**
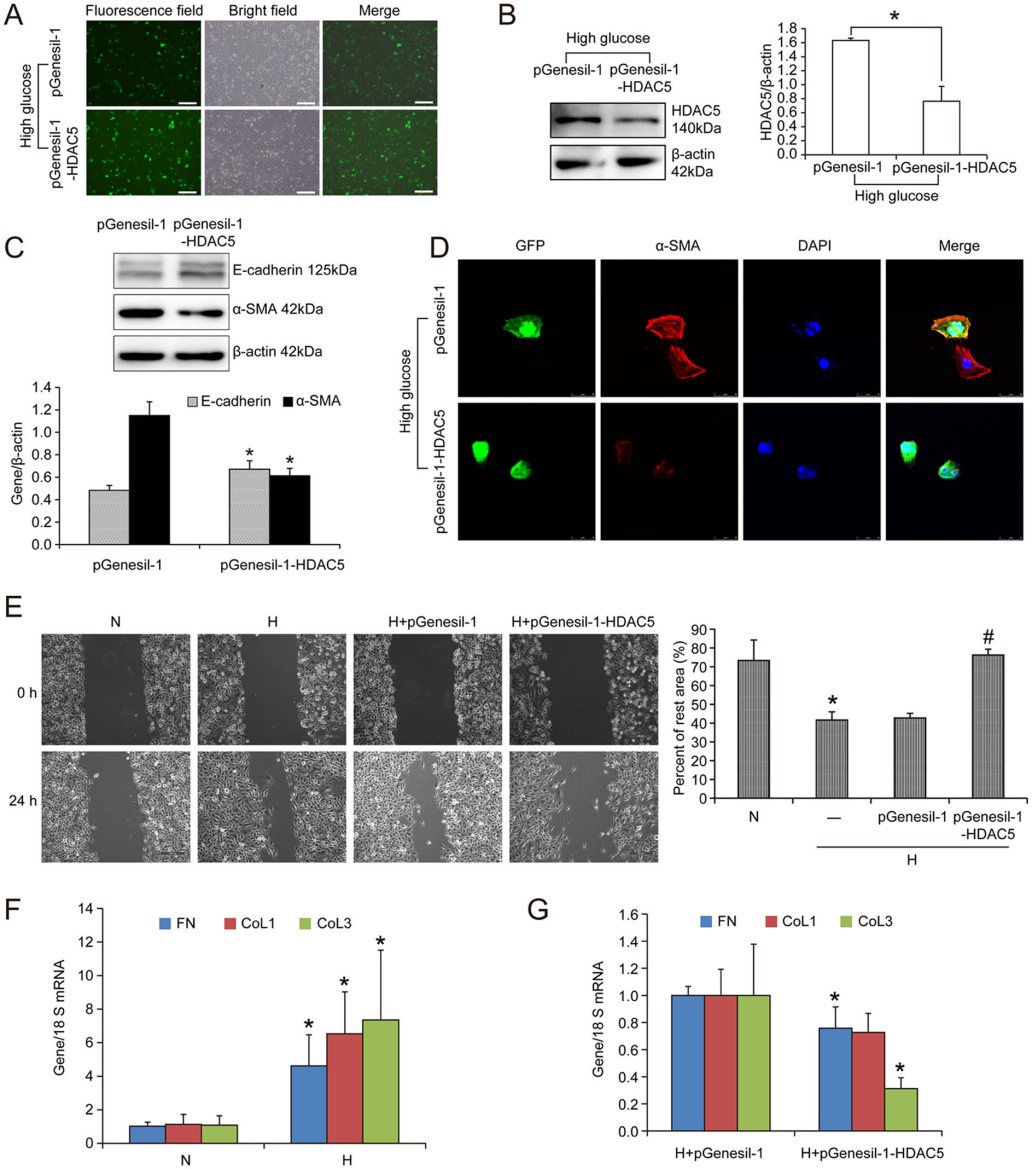
Knockdown of HDAC5 ameliorated high glucose-induced EMT of HK2 cells. **A** Transfection efficiency of pGenesil-1 and pGenesil-1-HDAC5 in HK2 cells. Bar: 50 μm. **B** Western blot and statistical analyses of HDAC5 expression in pGenesil-1 and pGenesil-1-HDAC5-transfected HK2 cells treated with high glucose. Data were representative of three independent experiments. *Means *P* < 0.05 versus pGenesil-1 group. **C** Western blot of E-cadherin and α-SMA expression in pGenesil-1 and pGenesil-1-HDAC5-transfected high glucose-cultured HK2 cells and statistical analyses. *Means *P* < 0.05 versus pGenesil-1 group. **D** Immunofluorescence of α-SMA in high glucose-treated HK2 cells transfected with pGenesil-1 and pGenesil-1-HDAC5. White arrows indicate successfully transfected HK2 cells. **E** Wound healing experiment of HK2 cells of normal glucose group (N), high glucose group (H), high glucose plus pGenesil-1 group (H + pGenesil-1), and high glucose plus pGenesil-1-HDAC5 group (H + pGenesil-1-HDAC5). *Means *P* < 0.05 versus N group, ^#^means *P* < 0.05 versus H + pGenesil-1 group. **F** Real-time PCR of fibronectin (FN), collagen 1 (Col 1), and collagen 3 (Col 3) mRNA expression in HK2 cells treated with normal glucose (N) and high glucose (H). Data were representative of three independent experiments. *Means *P* < 0.05 versus N group. **G** Real-time PCR of fibronectin (FN), collagen 1 (Col 1), and collagen 3 (Col 3) mRNA expression in high glucose-cultured HK2 cells transfected with pGenesil-1 and pGenesil-1-HDAC5. *Means *P* < 0.05 versus H + pGenesil-1 group.

To further elucidate the function of HDAC5 in renal tubular cells of diabetes mellitus, we knocked down HDAC5 in HK2 cells treated with high glucose using shRNA plasmid targeted at HDAC5 (pGenesil-1-HDAC5). It could be seen in Fig. 2A that transfection efficiency was more than 80% and HDAC5 was reduced by 53.24% in pGenesil-1-HDAC5-transfected HK2 cells compared with pGenesil-1-transfected HK2 cells (*P* < 0.05) (Fig. 2B). Then as seen in Fig. 2C, epithelial marker E-cadherin and mesenchymal marker α-SMA were regulated by HDAC5 knockdown in HK2 cells treated with high glucose. Statistical analysis revealed that E-cadherin was increased by 39.06% and α-SMA was decreased by 46.75% in high glucose-treated HK2 cell transfected with pGenesil-1-HDAC5 compared with those transfected with pGenesil-1 (*P* < 0.05). In line, immunofluorescence staining of α-SMA also presented that the high glucose-cultured HK2 cells successfully transfected with pGenesil-1 (indicated in GFP expression) showed the same α-SMA expression as those failed to be transfected with pGenesil-1 (no GFP expression). Differently, HK2 cells successfully transfected with pGenesil-1-HDAC5 plasmid showed evident green fluorescence (GFP expression), and the reduced α-SMA expression (red fluorescence) (Fig. 2D). Furthermore, cell migration experiment showed that 24 h-high glucose treatment enhanced cell migration ability of HK2 cells, which was prevented with pGenesil-1-HDAC5 transfection. In detail, the rest area was decreased by 43.20% in high glucose-treated HK2 cells versus normal glucose-cultured HK2 cells (*P* < 0.05). While pGenesil-1-HDAC5 transfection increased the rest area by 1.78 times compared with pGenesil-1 transfection in HK2 cells (*P* < 0.05) (Fig. 2E). Moreover, we detected ECM protein expression and found that high glucose-induced increased fibronectin, collagen 1, and collagen 3 mRNA. In detail, fibronectin, collagen 1, and collagen 3 mRNA were, respectively, increased by 4.53, 5.77, and 6.77 times (*P* < 0.05) (Fig. 2F). Again, high glucose-increased fibronectin, collagen 1, and collagen 3 mRNA were effectively avoided with HDAC5 knockdown (Fig. 2G). Therefore, these data suggested that HDAC5 played a key role in regulating EMT of renal tubular cells and ECM accumulation.

### TGF-β1 pathway was involved in HDAC5-regulated EMT of HK2 cells

**Fig. 3:**
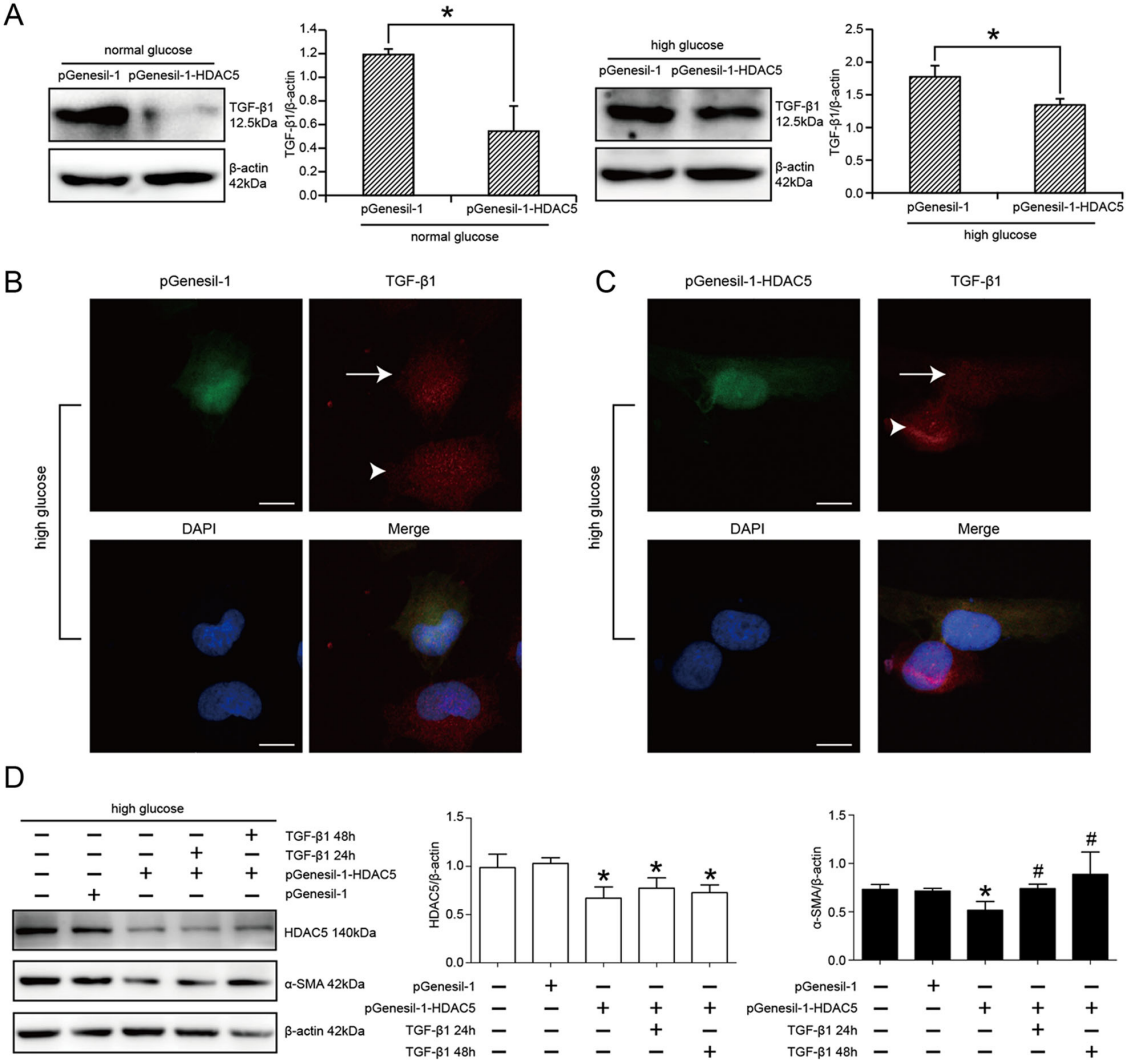
TGF-β1 pathway was involved in HDAC5-regulated EMT of HK2 cells. **A** Western blot and statistical analyses of TGF-β1 expression in normal glucose or high glucose-cultured HK2 cells transfected with pGenesil-1 and pGenesil-1-HDAC5. *n* = 3, *means *P* < 0.05 versus pGenesil-1 group. **B** Immunofluorescence of TGF-β1 in high glucose-cultured HK2 cells transfected with pGenesil-1. White arrow showed TGF-β1 expression in pGenesil-1-transfected cells. White triangle showed TGF-β1 expression in pGenesil-1-untransfected cells. Bar: 25 μm. **C** Immunofluorescence of TGF-β1 in high glucose-cultured HK2 cells transfected with pGenesil-1-HDAC5. White arrow showed weak TGF-β1 expression in pGenesil-1-HDAC5-transfected cells. White triangle showed evident TGF-β1 expression in pGenesil-1-HDAC5-untransfected cells. Bar: 25 μm. **D** Western blot and statistical analyses of the effect of TGF-β1 recombinant protein on pGenesil-1-HDAC5-reduced α-SMA expression in high glucose-cultured HK2 cells. *n* = 3, *means *P* < 0.05 versus pGenesil-1 group, ^#^means *P* < 0.05 versus pGenesil-1-HDAC5 group.

TGF-β1 pathway was the important signaling pathway to regulate EMT of renal tubular cells under the condition of hyperglycemia. We speculated that TGF-β1 pathway might be involved in HDAC5-reversed EMT of HK2, as the downstream targets of HDAC5. The results of western blot are illustrated in Fig. 3A that HDAC5 knockdown decreased TGF-β1 expression in both normal glucose-cultured HK2 cells and high glucose-cultured HK2 cells, especially in normal glucose-cultured cells. Statistical analysis confirmed TGF-β1 was, respectively, decreased by 54.36% and 24.35% in normal and high glucose-cultured HK2 cells transfected with pGenesil-1-HDAC5 versus pGenesil-1 (*P* < 0.05). In line with the results of western blot, immunofluorescence detection also showed that in pGenesil-1-transfected HK2 cells, there was no difference in TGF-β1 expression between transfected cells (with GFP expression) and untransfected cells (without GFP expression) (Fig. 3B). On the contrary, in pGenesil-1-HDAC5-transfected HK2 cells, the transfected cells indicated in GFP expression showed a decreased TGF-β1 expression compared with untransfected cells (Fig. 3C). Furthermore, exogenous TGF-β1 addition reversed the effect of HDAC5 knockdown on α-SMA in high glucose-treated HK2 cells. Statistical analysis revealed that α-SMA was, respectively, increased by 1.43 times and 1.72 times with 24 h-TGF-β1 treatment and 48 h-TGF-β1 treatment versus only pGenesil-1-HDAC5-transfected cells (*P* < 0.05) (Fig. 3D). Therefore, these above findings advised that HDAC5 knockdown ameliorated high glucose-induced EMT of renal tubular cells via TGF-β1 pathways.

### PI3K/Akt pathway mediated high glucose-induced HDAC5 upregulation in HK2 cells

**Fig. 4:**
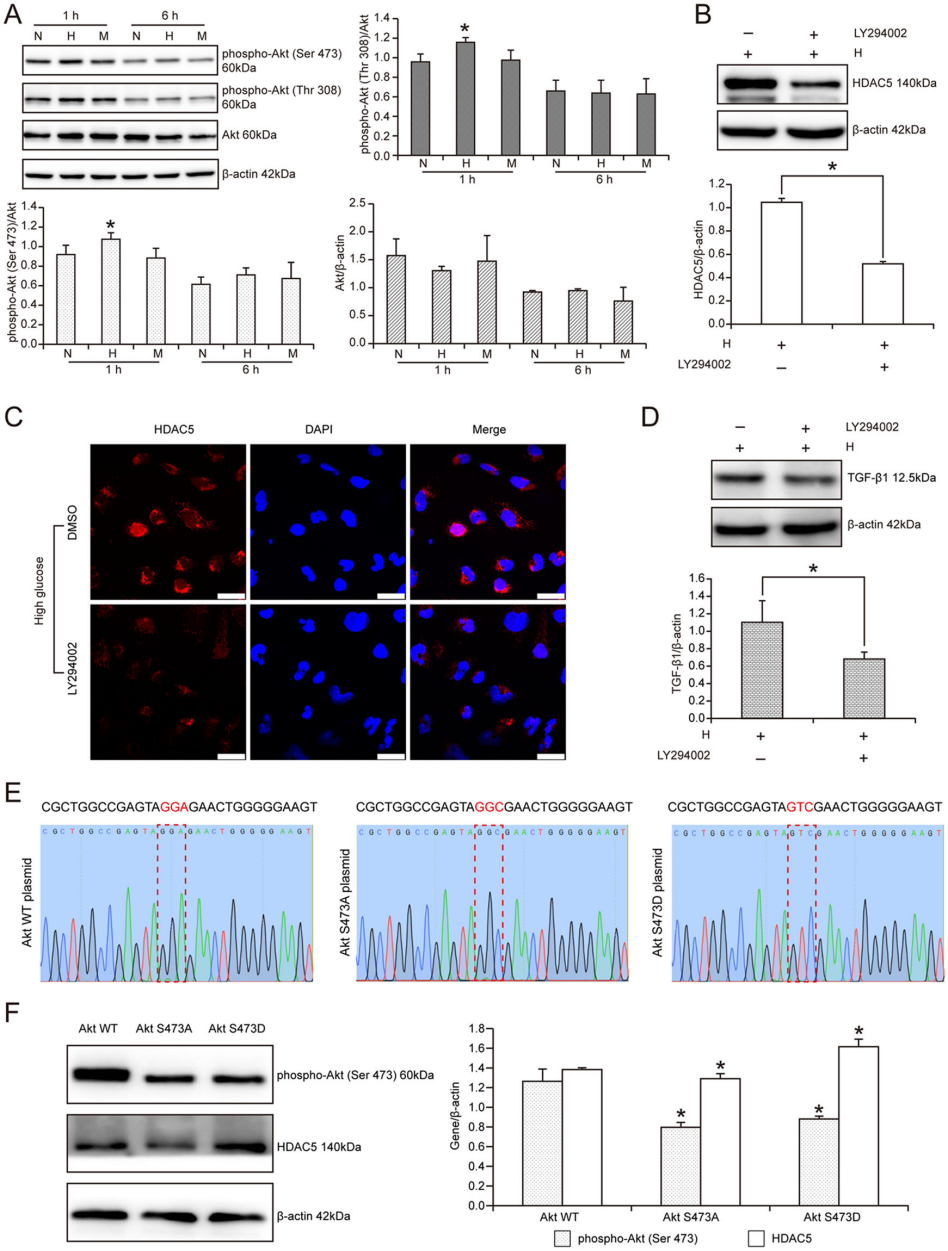
PI3K/Akt pathway mediated high glucose-induced HDAC5 upregulation in HK2 cells. Data were representative of three independent experiments. **A** Western blot and statistical analyses of phospho-Akt (Ser 473), phospho-Akt (Thr 308), and Akt in HK2 cells treated with high glucose for 1 h and 6 h. *Means *P* < 0.05 versus N group. **B** Western blot of HDAC5 and statistical analyses in HK2 cells treated with LY294002. *Means *P* < 0.05 versus H group. **C** Immunofluorescence of HDAC5 protein in high glucose-cultured HK2 cells treated with DMSO or LY294002. Bar: 25 μm. **D** Western blot of TGF-β1 and statistical analyses in HK2 cells treated with LY294002. *Means *P* < 0.05 versus H group. **E** DNA sequencing of the mutant site of Akt WT plasmid, Akt S473A plasmid and Akt S473D plasmid. **F** Western blot of phospho-Akt (Ser 473) and HDAC5 and statistical analyses in HK2 cells, respectively, transfected by Akt WT plasmid, Akt S473A plasmid and Akt S473D plasmid. *Means *P* < 0.05 versus Akt WT group.

Considering that PI3K/Akt pathway is the pivotal pathway to regulate renal tubular cell function in diabetes mellitus, we further explored the potential influence of PI3K/Akt pathway on high glucose-induced HDAC5 upregulation in HK2 cells. Firstly, we verified the quick activation of Akt pathway in HK2 cell stimulated with high glucose. Phospho-Akt (Ser 473) and phospho-Akt (Thr 308) were, respectively, increased by 1.20 times and 1.23 times with high glucose stimulation for 1 h versus normal glucose treatment in HK2 cells (*P* < 0.05) (Fig. 4A). Subsequently, inhibition of Akt pathway with LY294002 effectively prevented high glucose-caused HDAC5 upregulation. Compared with DMSO control treatment, LY294002 treatment decreased HDAC5 expression by 50.43% in high glucose-cultured HK2 cells (*P* < 0.05) (Fig. 4B). Also, the results of immunofluorescence were similar to the results of western blot. LY294002 inhibited high glucose-induced HDAC5 overexpression in HK2 cells (Fig. 4C). In turn, downstream target of HDAC5, TGF-β1 was also downregulated with the treatment of LY294002 in high glucose-cultured HK2 cells. In detail, TGF-β1 was decreased by 38.25% with LY294002 treatment (*P* < 0.05) (Fig. 4D).

To further determine the direct effect of Akt phosphorylation on HDAC5 expression in renal tubular cells, we, respectively, transfected HK2 cells with Akt wild-type plasmid pCMV3-Akt, Akt inactivated plasmid pCMV3-Akt (S473A), and Akt constitutively activated plasmid pCMV3-Akt (S473D) (Fig. 4E). The results were shown in Fig. 4F that compared with pCMV3-Akt plasmid-transfected HK2 cells, cells transfected with pCMV3-Akt (S473A) plasmid showed reduced HDAC5 expression. On the contrary, those cells transfected with pCMV3-Akt (S473D) plasmid presented enhanced HDAC5 expression. Statistical analysis revealed that HDAC5 expression was increased by 1.17 times with pCMV3-Akt (S473D) plasmid transfection versus pCMV3-Akt plasmid transfection (*P* < 0.05). Here, these findings suggested that Akt pathway activation was the upstream regulator of HDAC5 and TGF-β1 expression in high glucose-cultured HK2 cells.

### N6-methyladenosine demethylase inhibitor MA2 ameliorated high glucose-induced Akt pathway activation and HDAC5 expression in HK2 cells

**Fig. 5:**
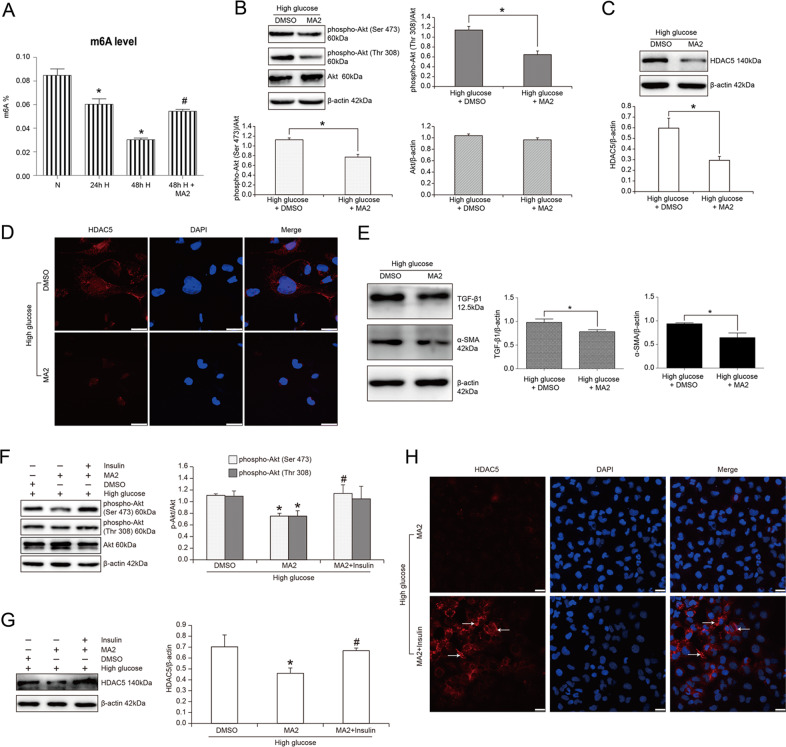
The effect of MA2 and insulin on phospho-Akt (Ser 473), phospho-Akt (Thr 308), Akt, HDAC5, TGF-β1, and α-SMA in HK2 cells. **A** Total m6A methylation level detection in HK2 cells treated with high glucose. *n* = 3, *means *P* < 0.05 versus N group. **B** Western blot and statistical analyses of phospho-Akt (Ser 473), phospho-Akt (Thr 308), and Akt in high glucose-cultured HK2 cells treated with DMSO or MA2. *n* = 3, *means *P* < 0.05 versus DMSO group. **C** Western blot and statistical analyses of HDAC5 in high glucose-cultured HK2 cells treated with DMSO or MA2. *n* = 3, *means *P* < 0.05 versus DMSO group. **D** Immunofluorescence of HDAC5 in high glucose-cultured HK2 cells treated with DMSO or MA2. Bar: 25 μm. **E** Western blot and statistical analyses of TGF-β1 and α-SMA in high glucose-cultured HK2 cells treated with DMSO or MA2. *n* = 3, * means *P* < 0.05 versus DMSO group. **F** Western blot and statistical analyses of phospho-Akt (Ser 473) and phospho-Akt (Thr 308) in HK2 cells. *n* = 3, *means *P* < 0.05 versus DMSO group, ^#^means *P* < 0.05 versus MA2 group. **G** Western blot and statistical analyses of HDAC5 in HK2 cells. *n* = 3, *means *P* < 0.05 versus DMSO group, ^#^means *P* < 0.05 versus MA2 group. **H** Immunofluorescence of HDAC5 expression in high glucose-cultured HK2 cells treated with MA2 or MA2 plus insulin. White arrows showed the positive expression of HDAC5. Bar: 25 μm.

N6-methyladenosine (m6A) RNA methylation is the most common RNA modification to regulate cell signaling conduction and gene expression. We first detected the level of total m6A mRNA methylation in high glucose-treated HK2 cells. The results were shown in Fig. 5A that high glucose reduced the level of total m6A RNA methylation in HK2 cells, which was prevented with 50 μmol/L MA2 treatment known as FTO (m6A RNA demethylase) inhibitor for 12 h. In detail, total m6A RNA methylation was, respectively, decreased by 28.95% and 64.25% with high glucose treatment for 24 h and 48 h versus normal glucose treatment (*P* < 0.05). Also, total m6A RNA methylation was enhanced by 1.79 times with MA2 treatment in 48 h-high glucose-cultured HK2 cells (*P* < 0.05). In addition, to further elucidate whether m6A RNA methylation was associated with Akt pathway activity and HDAC5 expression, we used MA2 to treat high glucose-cultured HK2 cells. Then Akt phosphorylation was significantly decreased with the treatment of MA2, followed by HDAC5 downregulation. Statistical analysis revealed that phospho-Akt (Ser 473) and phospho-Akt (Thr 308) were, respectively, decreased by 27.94% and 43.97% in high glucose-cultured HK2 cells with the treatment of MA2 compared to DMSO treatment (*P* < 0.05) (Fig. 5B). Similarly, HDAC5 expression was also reduced by 50.70% with MA2 treatment (*P* < 0.05) (Fig. 5C). Also, immunofluorescence detection showed the similar results to western blot that MA2 stimulation decreased HDAC5 protein expression (Fig. 5D). Again, TGF-β1 and EMT marker α-SMA were also decreased by the treatment of MA2 in high glucose-cultured HK2 cells. Statistical analysis revealed a 20.04% decrease of TGF-β1 and a 31.19% decrease of α-SMA in MA2-treated HK2 cells versus DMSO-treated cells (*P* < 0.05) (Fig. 5E). The above data suggested that N6-methyladenosine mRNA methylation level might mediate high glucose-induced Akt pathway activation, HDAC5 upregulation and EMT of renal tubular cells.

### Akt pathway agonist insulin prevented MA2-reduced HDAC5 expression in high glucose-treated HK2

In order to determine the direct regulation of m6A RNA modification on Akt pathway, as well as HDAC5 expression in high glucose-treated HK2, we pretreated HK2 cells with insulin (activator of PI3K/Akt pathway) and revealed that MA2-reduced HDAC5 expression was effectively reversed. Statistical analysis confirmed that the phospho-Akt (Ser 473) and phospho-Akt (Thr 308) were, respectively, enhanced by 1.60 times and 1.43 times in insulin plus MA2-treated HK2 cells compared with only MA2-treated cells (Fig. 5F). Similarly, HDAC5 expression was also increased by 1.45 times in insulin plus MA2-treated HK2 cells compared with only MA2-treated cells (*P* < 0.05) (Fig. 5G). The results of immunofluorescence were similar to those of western blot. Insulin treatment significantly increased HDAC5 expression, indicated in red fluorescence in MA2-stimulated HK2 cells (Fig. 5H). Taken together, these findings advised that Akt pathway really mediated MA2-decreased HDAC5 expression in high glucose-cultured HK2 cells.

### METTL14-regulated Akt pathway and HDAC5 expression in high glucose-treated HK2 cells

**Fig. 6:**
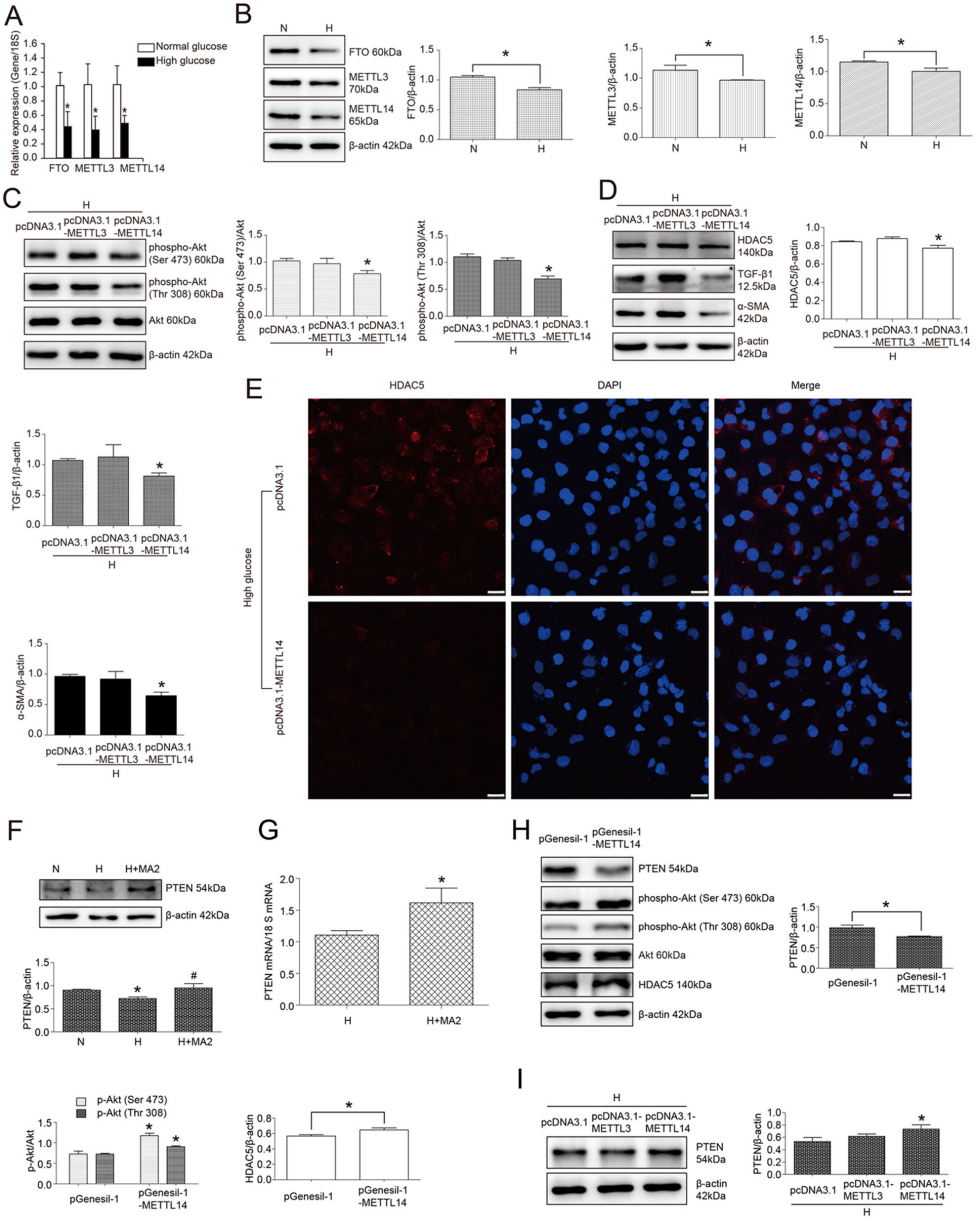
METTL14 upregulation reversed the effect of high glucose on phospho-Akt (Ser 473), phospho-Akt (Thr 308), HDAC5, TGF-β1, and α-SMA in HK2 cells through PTEN. Data were representative of three independent experiments. **A** Real-time PCR of FTO, METTL3 and METTL14 mRNA in HK2 cells treated with normal glucose or high glucose. *Means *P* < 0.05 versus normal glucose group. **B** Western blot and statistical analyses of FTO, METTL3, and METTL14 protein in HK2 cells treated with normal glucose or high glucose. *Means *P* < 0.05 versus normal glucose group. **C** Western blot and statistical analyses of phospho-Akt (Ser 473) and phospho-Akt (Thr 308) in high glucose-cultured HK2 cells transfected with pcDNA3.1, pcDNA3.1-METTL3, and pcDNA3.1-METTL14. *Means *P* < 0.05 versus pcDNA3.1 group. **D** Western blot and statistical analyses of HDAC5, TGF-β1, and α-SMA in high glucose-cultured HK2 cells transfected with pcDNA3.1, pcDNA3.1-METTL3, and pcDNA3.1-METTL14. *Means *P* < 0.05 versus pcDNA3.1 group. **E** Immunofluorescence of HDAC5 protein in high glucose-cultured HK2 cells transfected with pcDNA3.1 and pcDNA3.1-METTL14. Bar: 25 μm. **F** Western blot and statistical analyses of PTEN in HK2 cells treated with normal glucose (N), high glucose (H), and high glucose plus MA2 (H + MA2). *Means *P* < 0.05 versus N group, ^#^means *P* < 0.05 versus H group. **G** Real-time PCR of PTEN mRNA in HK2 cells treated with high glucose or high glucose plus MA2. *Means *P* < 0.05 versus H group. **H** Western blot and statistical analyses of *P*TEN, phospho-Akt (Ser 473), phospho-Akt (Thr 308), and HDAC5 in HK2 cells transfected with pGenesil-1 and pGenesil-1-METTL14. *Means *P* < 0.05 versus pGenesil-1 group. **I** Western blot and statistical analyses of PTEN in high glucose-cultured HK2 cells transfected with pcDNA3.1, pcDNA3.1-METTL3, and pcDNA3.1-METTL14. *Means *P* < 0.05 versus pcDNA3.1 group.

Considering that m6A RNA modification was regulated by methylase and demethylase, we further explored which enzyme of m6A RNA modification was involved in high glucose-induced Akt pathway activation and HDAC5 upregulation in HK2 cells. Then we first detected FTO, METTL3, and METTL14 mRNA expression by the method of real-time PCR in high glucose-stimulated cells. As illustrated in Fig. 6A, high glucose treatment significantly decreased FTO, METTL3, and METTL14 mRNA expression. In detail, FTO, METTL3, and METTL14 mRNA were, respectively, decreased by 56.68%, 61.71%, and 52.69% with high glucose treatment in HK2 cells compared with normal glucose treatment (*P* < 0.05). Similarly, western blot results also showed that high glucose reduced FTO, METTL3, and METTL14 at the level of protein in HK2 cells. Statistical analysis revealed that FTO, METTL3, and METTL14 protein were, respectively, decreased by 20.36%, 14.72%, and 12.80% with high glucose treatment versus normal glucose treatment (*P* < 0.05) (Fig. 6B).

In view of the decreased m6A RNA modification level in high glucose-cultured HK2 cells, we speculated that methylase METTL3 and METTL14 downregulation might be the main reason to affect m6A RNA modification and Akt pathway, and demethylase FTO downregulation might be compensatory. Hence, we upregulated METTL3 and METTL14 expression in high glucose-cultured HK2 cells and revealed that METTL14 not METTL3 overexpression reversed the effect of high glucose on Akt phosphorylation. Compared with pcDNA3.1 transfection, phospho-Akt (Ser 473), and phospho-Akt (Thr 308) were, respectively, reduced by 18.07% and 34.71% with pcDNA3.1-METTL14 transfection (*P* < 0.05). However, there was no difference in phospho-Akt (Ser 473) and phospho-Akt (Thr 308) expression between cells of pcDNA3.1 transfection group and those of pcDNA3.1-METTL3 transfection group (*P* > 0.05) (Fig. 6C). Subsequently, HDAC5 protein was also downregulated by 15.80% with the transfection of METTL14 expression plasmid versus blank plasmid in high glucose-cultured HK2 cells (*P* < 0.05), not METTL3. In line, EMT-associated proteins TGF-β1 and α-SMA were also decreased by pcDNA3.1-METTL14 plasmid transfection in high glucose-cultured HK2 cells. In detail, TGF-β1 and α-SMA were, respectively, reduced by 23.99% and 33.05% in pcDNA3.1-METTL14-transfected HK2 cells versus pcDNA3.1-transfected cells (*P* < 0.05) (Fig. 6D). In addition, immunofluorescence detection also revealed that HDAC5 expression was significantly inhibited by pcDNA3.1-METTL14 plasmid transfection in HK2 cells (Fig. 6E). Taken together, it was suggested that METTL14-mediated m6A RNA modification affected Akt pathway, HDAC5, TGF-β1, and α-SMA in high glucose-stimulated HK2 cells.

### PTEN mediated MA2 and METTL14-inhibited Akt pathway activation in high glucose-cultured HK2 cells

Considering that m6A RNA modification affected RNA degradation or protein translation, and not directly affected protein phosphorylation, we speculated that MA2 and METTL14 might regulate Akt signaling pathway by affecting the upstream regulator of Akt. In our previous study, PTEN was revealed to be the crucial regulator of Akt pathway in renal tubular cells of diabetic kidney disease. Here, we first detected the effect of MA2 on PTEN expression in high glucose-cultured HK2 cells and the results showed that MA2 significantly increased PTEN expression at the level of protein. Statistical analysis revealed that PTEN protein was increased by 1.32 times by the treatment of MA2 in HK2 cells (*P* < 0.05) (Fig. 6F). Similarly, the results of real-time PCR showed that PTEN mRNA level was also increased by 1.46 times in MA2-treated HK2 cells (*P* < 0.05) (Fig. 6G). Then, in normal glucose-cultured HK2 cells, METTL14 shRNA plasmid transfection caused a 21.81% decrease of PTEN expression, similar to the effect of high glucose on PTEN protein. Accordingly, phospho-Akt (Ser 473) and phospho-Akt (Thr 308) were, respectively, enhanced by 1.23 times and 1.56 times with pGenesil-1-METTL14 transfection versus pGenesil-1 transfection (*P* < 0.05). Subsequently, HDAC5 protein expression was also increased (Fig. 6H). Furthermore, we overexpressed METTL3 and METTL14 in high glucose-cultured HK2 cells and found that pcDNA3.1-METTL14 not pcDNA3.1-METTL3 significantly increased PTEN expression by 1.38 times versus pcDNA3.1 (*P* < 0.05). There was no evident difference in PTEN expression between pcDNA3.1-transfected HK2 cells and pcDNA3.1-METTL3-transfected cells (Fig. 6I). Therefore, it was suggested that PTEN upregulation was involved in MA2 and METTL14-caused Akt pathway inactivation and HDAC5 downregulation in HK2 cells.

### HDACi TSA treatment decreased HDAC5 expression, EMT, and ECM accumulation in renal tubular cells of diabetic mice

**Fig. 7:**
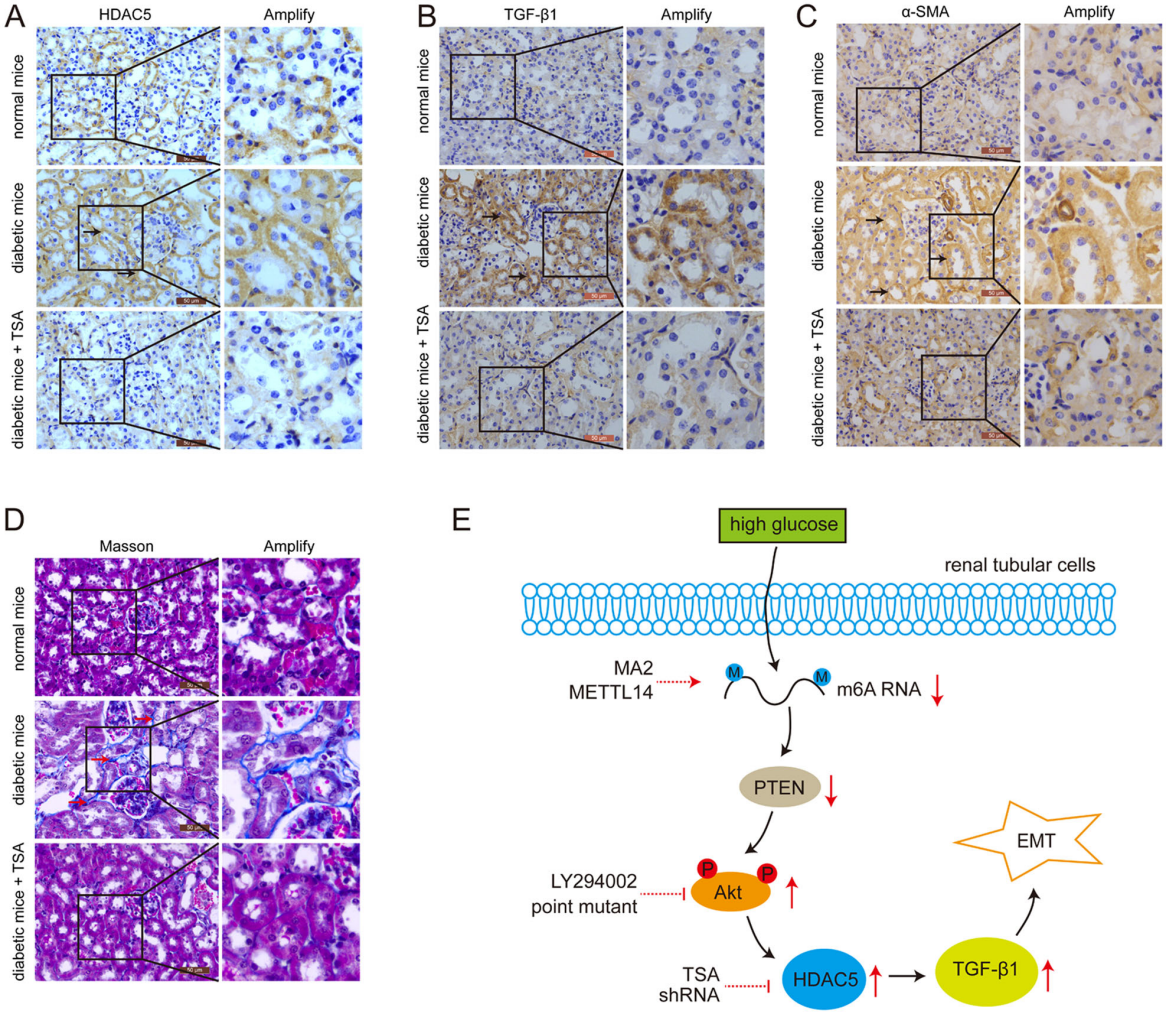
The effect of TSA on HDAC5, TGF-β1, α-SMA, and ECM in the kidney of diabetic mice (*n* = 6). **A** Immunohistochemistry of HDAC5 in the kidney of normal mice, diabetic mice and diabetic mice treated with TSA. Black arrows showed the positive signal of HDAC5. **B** Immunohistochemistry of TGF-β1 in the kidney of normal mice, diabetic mice and diabetic mice treated with TSA. Black arrows showed the positive signal of TGF-β1. **C** Immunohistochemistry of α-SMA in the kidney of normal mice, diabetic mice and diabetic mice treated with TSA. Black arrows showed the positive signal of α-SMA. **D** Masson staining of the kidney of normal mice, diabetic mice and diabetic mice treated with TSA. Red arrows showed ECM accumulation. **E** Model of the effect of m6A RNA methylation-regulated PTEN/PI3K/Akt signaling pathway on HDAC5-mediated epithelial–mesenchymal transition in high glucose-treated renal tubular cells.

Again, we further explored the in vivo effect of HDACs inhibitor on HDAC5 expression, EMT and ECM deposit in the kidneys of diabetic mice. It could be seen in Fig. 7A that HDAC5 was effectively decreased in the renal tubular cells of trichostatin A (TSA)-administrated diabetic mice. In line, overexpression of TGF-β1 in the kidneys of diabetic mice was significantly inhibited with TSA treatment (Fig. 7B). Furthermore, immunohistochemistry results also revealed that EMT marker α-SMA was markedly enhanced in the renal tubular cells of diabetic mice compared with those of normal mice, which was avoided with the administration of TSA (Fig. 7C). Then Masson staining showed that the cumulative ECM in renal interstitium of diabetic mice was effectively prevented in the kidneys of TSA-treated diabetic mice (Fig. 7D). Therefore, these findings suggested that HDACi was the promising drug to ameliorate EMT of renal tubular cells and ECM deposit of diabetic kidney disease.

## Discussion

In the present study, we first revealed the increased expression of HDAC5 in renal tubular cells and interstitium of diabetic mice and UUO mice, especially UUO mice. Also, HDAC5 overexpression promoted EMT of renal tubular cells, indicated in E-cadherin downregulation and α-SMA upregulation. Similarly, Choi et al. found that piceatannol suppressed renal fibrosis, shown as decreased ECM protein, reduced connective tissue growth factor (CTGF) and α-SMA in UUO kidneys, accompanied with reduced HDAC4 and HDAC5 protein expression^16^. As well known, EMT was the important characteristic of tumor to regulate invasion and metastasis, and HDAC5 was also reported to mediate EMT of tumor cells. In non-small cell lung cancer, the loss of miR-589-5p led to HDAC5 expression upregulation, regulating cell cycle and EMT-related genes^17^. As well, knockdown of HDAC5 by siRNA technology in glioma cells suppressed the doxorubicin-induced EMT^18^. Recently, Jaquva et al. revealed that HDAC5 overexpression in multiple urothelial carcinoma (UC) cell lines decreased cell proliferation, however in VM-Cub-1 cell line HDAC5 overexpression induced a dramatic EMT change, with the weak effect of HDAC4 on cell phenotypes^19^. Taken together it is suggested that HDAC5 is a promising target to ameliorate diabetic kidney disease via regulating EMT.

TGF-β1 is well known as pro-fibrosis factor and previously reported to increase in renal tubular cells of diabetes mellitus^20^. Our findings revealed that TGF-β1 was involved in HDAC5-regulated EMT in renal tubular cells. As so far, there was no report on the direct relationship between HDAC5 and TGF-β1, whereas HDACs inhibitors (HDACi) or other HDACs members were proven to affect TGF-β1 expression in fibrosis models including renal fibrosis. Suberoylanilide hydroxamic acid (SAHA) alleviated live fibrosis by suppressing TGF-β1 pathway in the fibrotic rats^21^. In line, Yang et al. revealed that FK228 known as a selective inhibitor of class I HDACs significantly suppressed renal interstitial fibrosis via Smad and non-Smad pathways in a murine model of UUO^22^. Additionally, the mesothelial cells (MCs) isolated from effluent of peritoneal dialysis (PD) patients were treated with MS-275, a HDAC1–3 inhibitor, and downregulated mesenchymal markers including TGF-β1 to promote EMT reversal. Further genetic silencing experiments confirmed that HDAC1 played a major role in EMT reversal^23^. Combined with these findings, our experiments suggested that TGF-β1 was the important downstream target of HDAC5-regulated EMT in diabetic kidney disease. Here, we also found knockdown of HDAC5 in normal glucose-cultured HK2 cells led to a more significant downregulation of TGF-β1 than high glucose-cultured cells. Considering that hyperglycemia had been proven to activate TGF-β1 pathway in renal tubular cells of diabetic kidney disease^24^, we speculated that hyperglycemia environment compromised the inhibitory effect of HDAC5 knockdown on TGF-β1 in HK2 cells.

Cell signaling pathway mediated multiple functions of cells including proliferation, migration, apoptosis, autophagy, and metabolism. PI3K/Akt pathway has been proven to be activated in renal tubular cells of diabetes mellitus^25^. Here, we also revealed that PI3K/Akt pathway regulated HDAC5 expression and inhibition of PI3K/Akt pathway ameliorated high glucose-induced EMT of renal tubular cells by the downregulation of HDAC5. Similar to our findings, in L6 myocytes decreased Akt phosphorylation resulted from low pH (pH 7.0) stimulation caused the reduced nuclear content of HDAC5^26^. Also, Pietruczuk et al. found that two inhibitors of PI3K/Akt pathway, wortmannin and SC66, evidently attenuated the phosphorylation of HDAC5 and nuclear content in IGF-1-induced vascular smooth muscle cells (VSMCs)^27^. Summarily, PI3K/Akt pathway is the upstream signaling pathway to regulate HDAC5 expression in renal tubular cells of diabetic kidney disease.

Inspired by Liu et al.’s finding that m6A mRNA methylation regulated Akt activity in endometrial cancer cells^12^, we further explored the potential role of m6A mRNA methylation level in high glucose-induced Akt pathway activation and HDAC5 expression in renal tubular cells. Similar to the previous studies that m6A RNA methylation was decreased in peripheral blood RNA from type 2 diabetes mellitus (T2DM) patients compared with control group^28^, we also confirmed high glucose-induced m6A RNA methylation level downregulation in vitro-cultured HK2 cells. Subsequently, m6A demethylase inhibitor MA2 or overexpression of METTL14 inhibited Akt pathway activation followed by HDAC5 downregulation in high glucose-cultured HK2 cells. Recently, the regulation of m6A modification on Akt pathway was also revealed in gastric cancer cells. Zhang et al. confirmed that in gastric cancer cell lines HGC‐27 and MGC803, m6A modification suppression via METTL14 knockdown promoted cell proliferation and invasion by activating Wnt and PI3K-Akt signaling. On the contrary, m6A elevation via FTO knockdown reversed these phenotypes^29^. Therefore, high glucose-caused m6A RNA methylation level downregulation activated Akt pathway in renal tubular cells, leading to HDAC5 expression abnormality.

PTEN was known as the negative regulator of Akt pathway and proven to be regulated by MA2 and METTL14 in HK2 cells in this study. PTEN mediated MA2 and METTL14 overexpression-caused Akt signaling pathway inactivation in HK2 cells. Similarly, the relationship between PTEN and m6A modifications was also reported in the human acute myeloid leukemia MOLM-13 cell line. Single-nucleotide-resolution mapping of mA coupled with ribosome profiling revealed that mA modification promoted the PTEN mRNA translation^30^. Also, Wang et al. found that in renal clear cell carcinoma METTL14 mRNA was likely to regulate PTEN mRNA via changing m6A RNA modification level^31^. Therefore, it was suggested that PTEN might be the target of m6A RNA modification in HK2 cells affecting Akt pathway.

All in all, hyperglycemia increases HDAC5 expression in renal tubular cells of diabetic kidney, leading to epithelial–mesenchymal transition. TGF-β1 upregulation mediates HDAC5-caused epithelial–mesenchymal transition of renal tubular cells. N6-methyladenosine RNA methylation suppression-induced Akt pathway activation regulates HDAC5 expression in high glucose-cultured renal tubular cells. PTEN mediates m6A RNA modification-regulated Akt pathway activity in HK2 cells (Fig. 7E).

## Supplementary information

uncropped Fig.1-2

uncropped Fig.3

uncropped Fig.4

uncropped Fig.5

uncropped Fig.6

## Data Availability

All data generated in the study are included in this article.

## References

[1] Gnudi L, Coward RJM, Long DA (2016). Diabetic nephropathy: perspective on novel molecular mechanisms. Trends Endocrinol. Metab..

[2] Zhao Y (2017). MiR-30c protects diabetic nephropathy by suppressing epithelial-to-mesenchymal transition in db/db mice. Aging Cell.

[3] Hills CE, Squires PE (2011). The role of TGF-beta and epithelial-to mesenchymal transition in diabetic nephropathy. Cytokine Growth Factor Rev..

[4] Dong W (2017). Sodium butyrate activates NRF2 to ameliorate diabetic nephropathy possibly via inhibition of HDAC. J. Endocrinol..

[5] Hadden MJ, Advani A (2018). Histone deacetylase inhibitors and diabetic kidney disease. Int J. Mol. Sci..

[6] Yoshikawa M, Hishikawa K, Marumo T, Fujita T (2007). Inhibition of histone deacetylase activity suppresses epithelial-to-mesenchymal transition induced by TGF-beta1 in human renal epithelial cells. J. Am. Soc. Nephrol..

[7] Mathias RA, Guise AJ, Cristea IM (2015). Post-translational modifications regulate class IIa histone deacetylase (HDAC) function in health and disease. Mol. Cell Proteom..

[8] Wang X (2014). Histone deacetylase 4 selectively contributes to podocyte injury in diabetic nephropathy. Kidney Int..

[9] LoRusso PM (2016). Inhibition of the PI3K/AKT/mTOR pathway in solid tumors. J. Clin. Oncol..

[10] Hao J (2014). Phosphorylation of PRAS40-Thr246 involved in renal lipid accumulation of diabetes. J. Cell Physiol..

[11] Xue M (2018). Triptolide attenuates renal tubular epithelial-mesenchymal transition via the MiR-188-5p-mediated PI3K/AKT pathway in diabetic kidney disease. Int. J. Biol. Sci..

[12] Liu J (2018). m(6)A mRNA methylation regulates AKT activity to promote the proliferation and tumorigenicity of endometrial cancer. Nat. Cell Biol..

[13] Wei W, Ji X, Guo X, Ji S (2017). Regulatory role of N(6) -methyladenosine (m(6) A) methylation in RNA processing and human diseases. J. Cell Biochem..

[14] Jun H (2009). In vivo and in vitro effects of SREBP-1 on diabetic renal tubular lipid accumulation and RNAi-mediated gene silencing study. Histochem. Cell Biol..

[15] Du W (2019). STAT3 phosphorylation mediates high glucose-impaired cell autophagy in an HDAC1-dependent and -independent manner in Schwann cells of diabetic peripheral neuropathy. FASEB J..

[16] Choi SY (2016). Piceatannol attenuates renal fibrosis induced by unilateral ureteral obstruction via downregulation of histone deacetylase 4/5 or p38-MAPK signaling. PLoS One.

[17] Liu C (2017). Hypermethylation of miRNA-589 promoter leads to upregulation of HDAC5 which promotes malignancy in non-small cell lung cancer. Int. J. Oncol..

[18] Liu Q (2015). Formononetin sensitizes glioma cells to doxorubicin through preventing EMT via inhibition of histone deacetylase 5. Int. J. Clin. Exp. Pathol..

[19] Jaguva-Vasudevan AA (2019). HDAC5 Expression in Urothelial Carcinoma Cell Lines Inhibits Long-Term Proliferation but Can Promote Epithelial-to-Mesenchymal Transition. Int. J. Mol. Sci..

[20] Hao J (2011). PI3K/Akt pathway mediates high glucose-induced lipogenesis and extracellular matrix accumulation in HKC cells through regulation of SREBP-1 and TGF-beta1. Histochem. Cell Biol..

[21] Wang Y (2018). Histone deacetylase inhibitor suberoylanilide hydroxamic acid alleviates liver fibrosis by suppressing the transforming growth factor-beta1 signal pathway. Hepatobiliary Pancreat. Dis. Int..

[22] Yang M (2019). Inhibition of class I HDACs attenuates renal interstitial fibrosis in a murine model. Pharm. Res..

[23] Rossi L (2018). HDAC1 inhibition by MS-275 in mesothelial cells limits cellular invasion and promotes MMT reversal. Sci. Rep..

[24] Murphy M (2008). IHG-1 amplifies TGF-beta1 signaling and is increased in renal fibrosis. J. Am. Soc. Nephrol..

[25] Zhao S (2012). PI3K/Akt pathway mediates high glucose-induced lipid accumulation in human renal proximal tubular cells via spliced XBP-1. J. Cell Biochem..

[26] Genders AJ, Martin SD, McGee SL, Bishop DJ (2019). A physiological drop in pH decreases mitochondrial respiration, and HDAC and Akt signaling, in L6 myocytes. Am. J. Physiol. Cell Physiol..

[27] Pietruczuk P, Jain A, Simo-Cheyou ER, Anand-Srivastava MB, Srivastava AK (2019). Protein kinase B/AKT mediates insulin-like growth factor 1-induced phosphorylation and nuclear export of histone deacetylase 5 via NADPH oxidase 4 activation in vascular smooth muscle cells. J. Cell Physiol..

[28] Shen F (2015). Decreased N(6)-methyladenosine in peripheral blood RNA from diabetic patients is associated with FTO expression rather than ALKBH5. J. Clin. Endocrinol. Metab..

[29] Zhang C (2019). Reduced m6A modification predicts malignant phenotypes and augmented Wnt/PI3K-Akt signaling in gastric cancer. Cancer Med..

[30] Vu LP (2017). The N(6)-methyladenosine (m(6)A)-forming enzyme METTL3 controls myeloid differentiation of normal hematopoietic and leukemia cells. Nat. Med..

[31] Wang Q (2019). Identification of METTL14 in kidney renal clear cell carcinoma using bioinformatics analysis. Dis. Markers.

