# Supplementary figures and images for: METTL14-regulated PI3K/Akt signaling pathway via PTEN affects HDAC5-mediated epithelial–mesenchymal transition of renal tubular cells in diabetic kidney disease
Source: Cell Death Dis. 2021 Jan 4;12(1):32. doi: 10.1038/s41419-020-03312-0 (PMC7791055; doi:10.1038/s41419-020-03312-0)

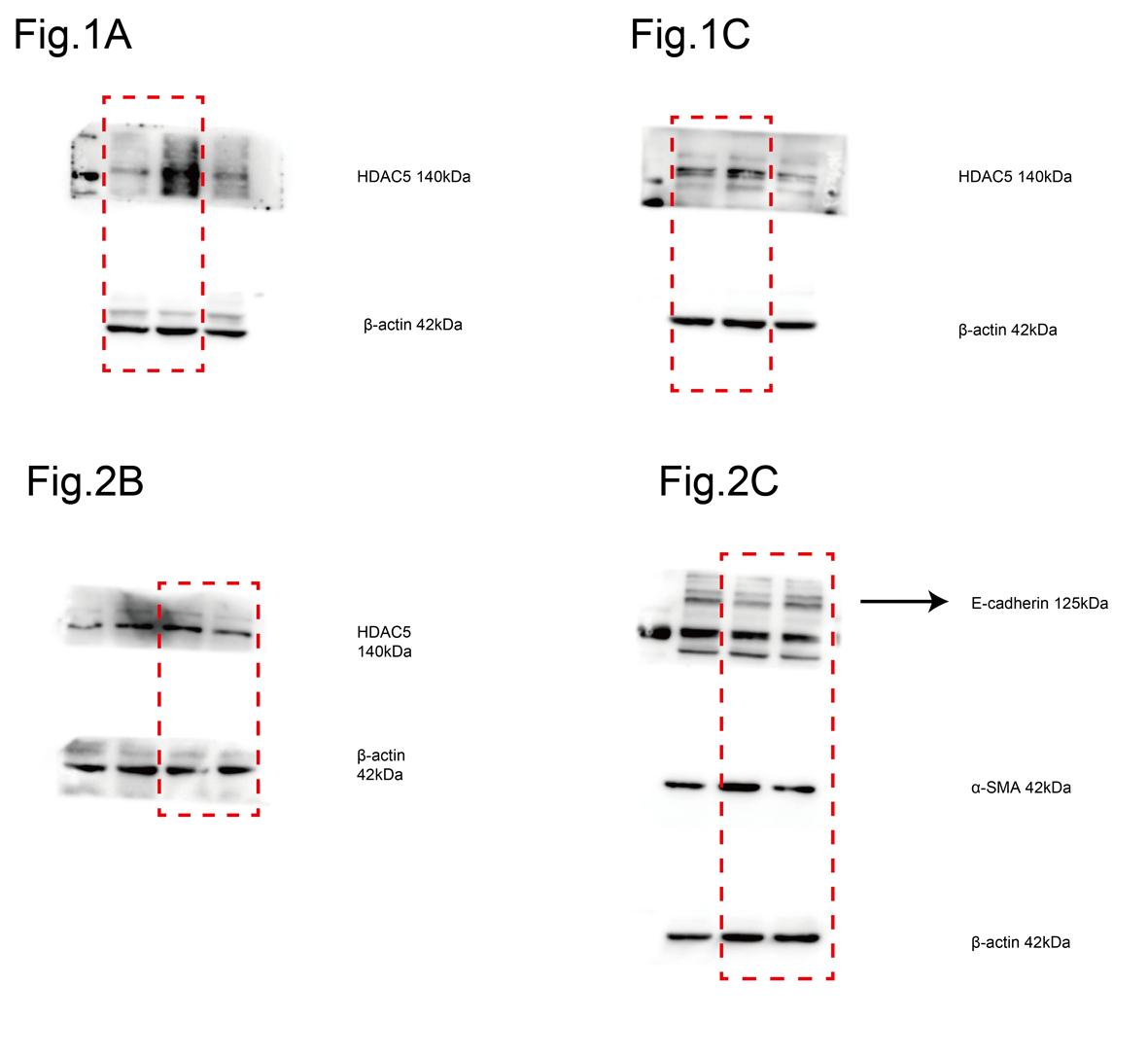

Supplement: Supplementary file 1 — uncropped Fig.1-2 [file 41419_2020_3312_MOESM1_ESM.tif]

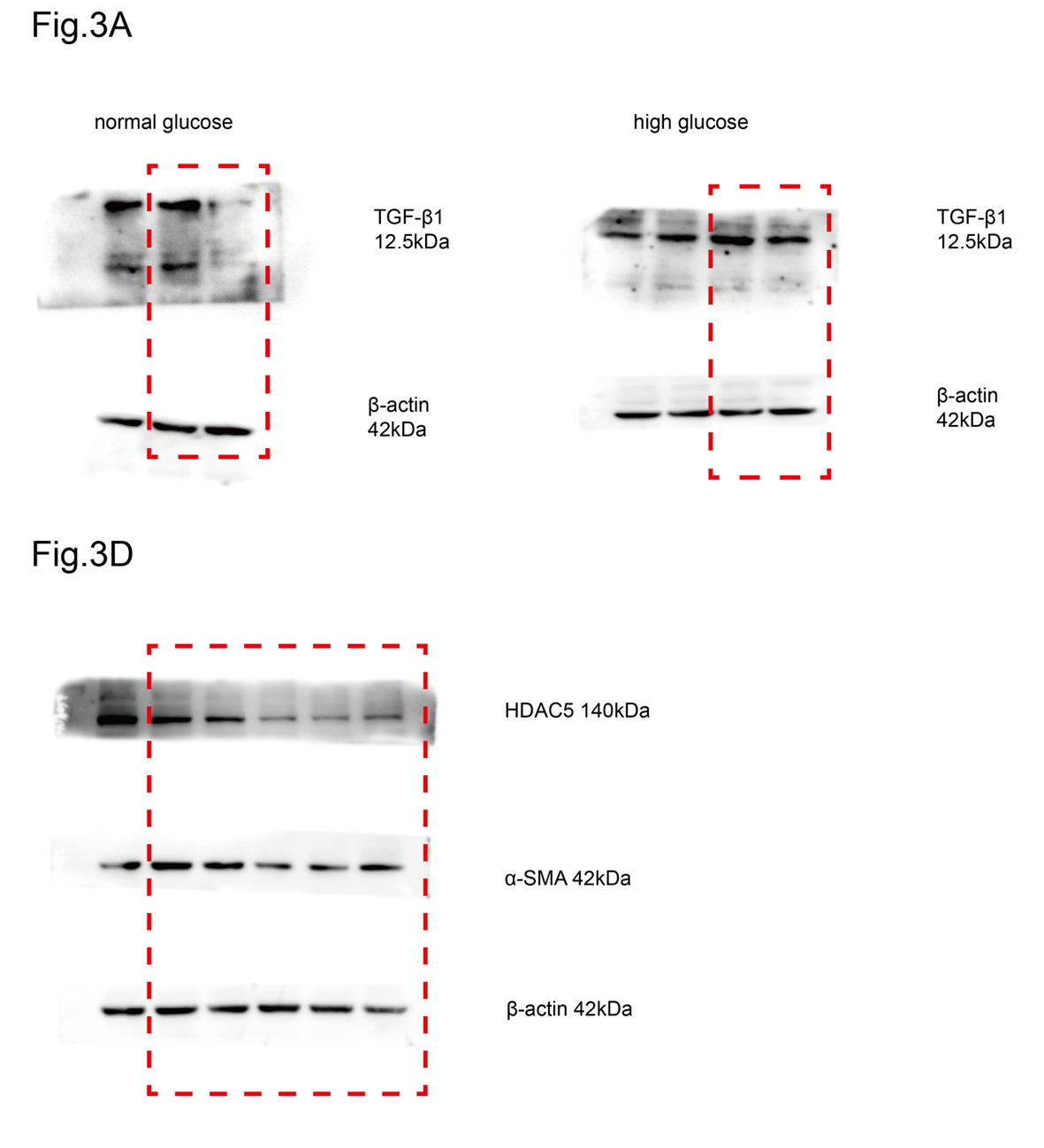

Supplement: Supplementary file 2 — uncropped Fig.3 [file 41419_2020_3312_MOESM2_ESM.tif]

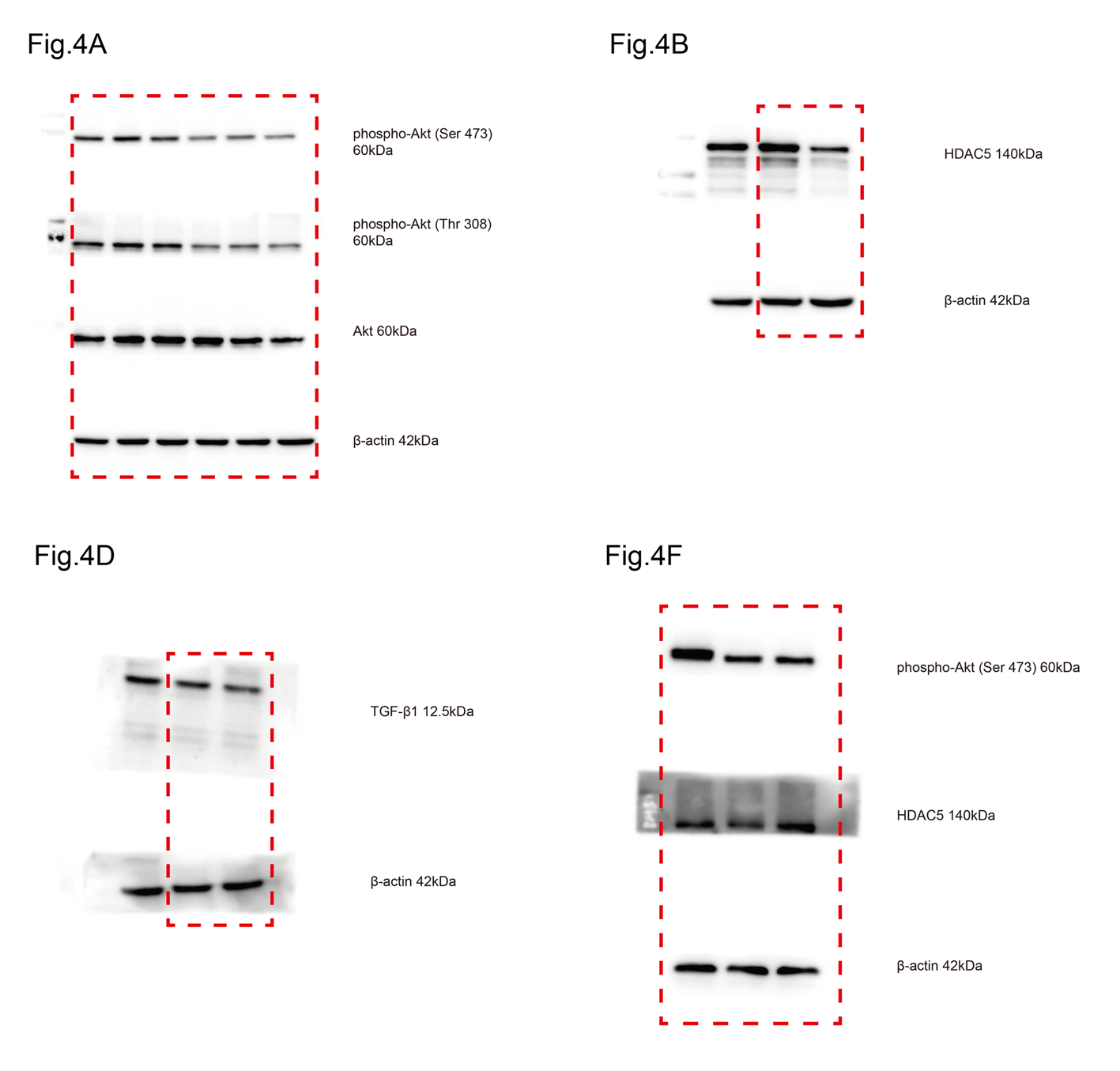

Supplement: Supplementary file 3 — uncropped Fig.4 [file 41419_2020_3312_MOESM3_ESM.tif]

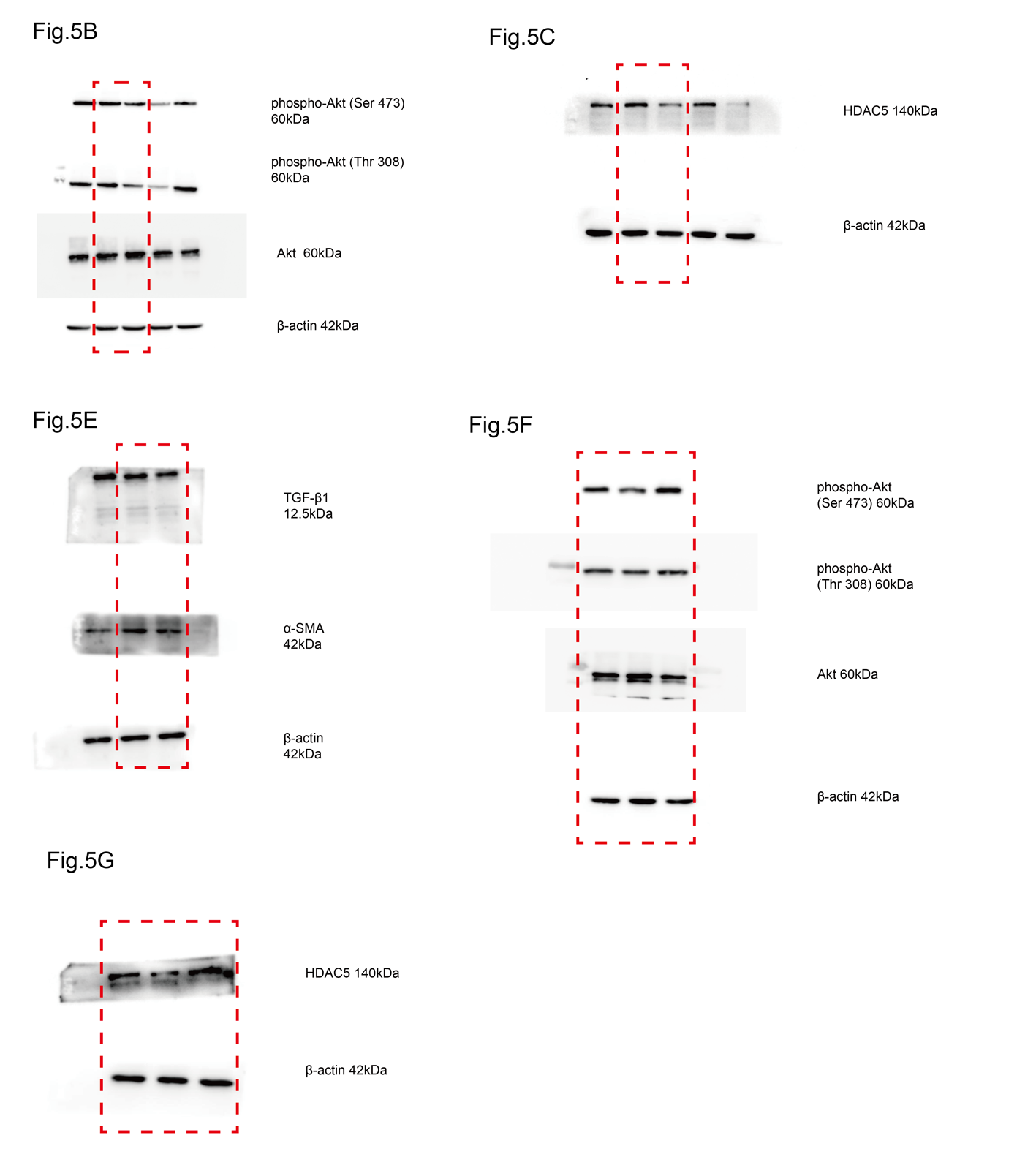

Supplement: Supplementary file 4 — uncropped Fig.5 [file 41419_2020_3312_MOESM4_ESM.tif]

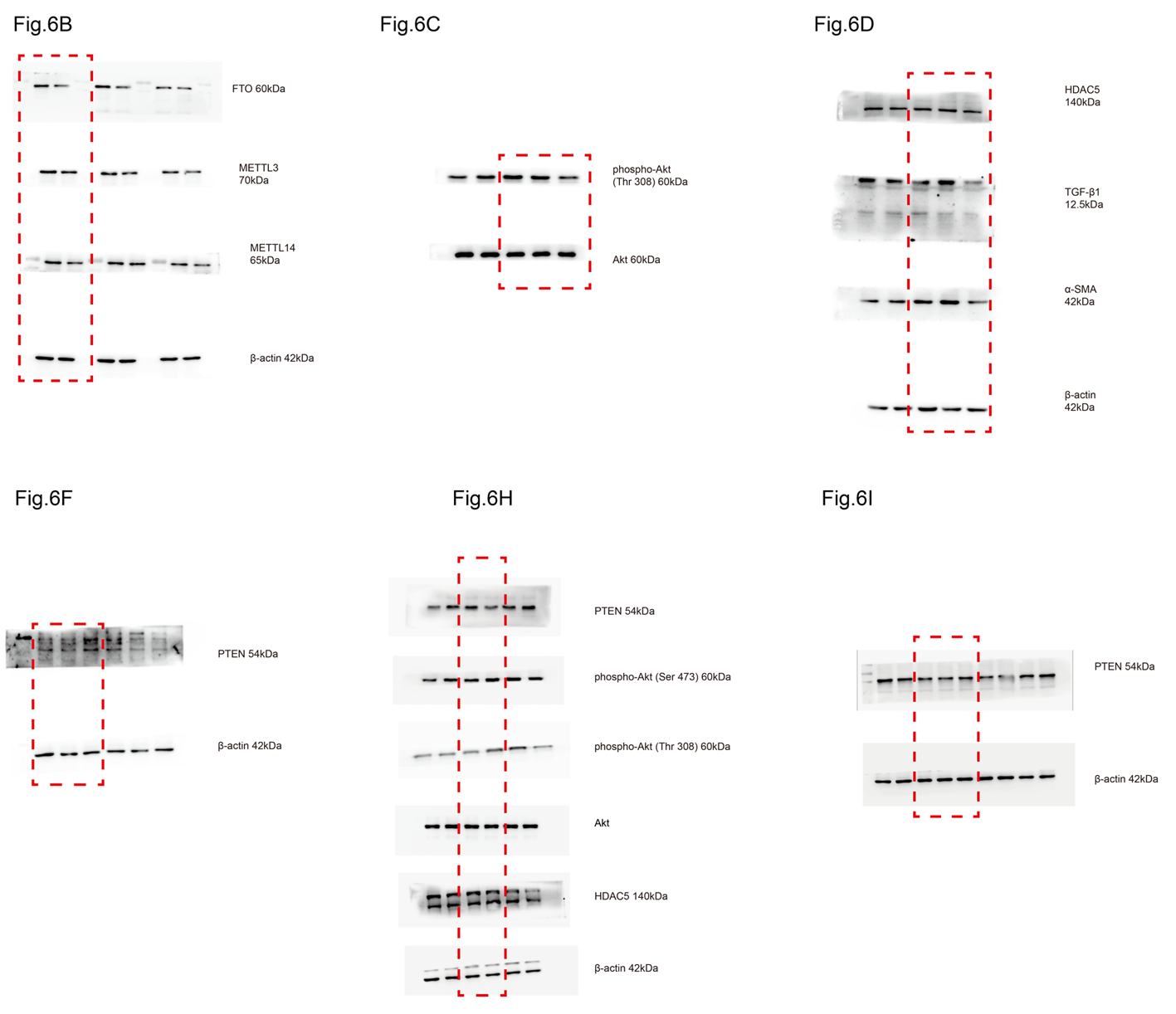

Supplement: Supplementary file 5 — uncropped Fig.6 [file 41419_2020_3312_MOESM5_ESM.tif]
